# Systematic comparison of nonviral gene delivery strategies for efficient co-expression of two transgenes in human mesenchymal stem cells

**DOI:** 10.1186/s13036-023-00394-0

**Published:** 2023-12-07

**Authors:** Tyler Kozisek, Luke Samuelson, Andrew Hamann, Angela K. Pannier

**Affiliations:** Department of Biological Systems Engineering, University of Nebraska-Lincoln, Lincoln, NE 68583-0900 USA

**Keywords:** Human mesenchymal stem cells, Transfection, Lipid-mediated transfection, Polymer-mediated transfection, 2A peptide sequence, IRES, Nonviral gene delivery, Co-transfection, Bicistronic

## Abstract

**Background:**

Human mesenchymal stem cells (hMSCs) are being researched for cell-based therapies due to a host of unique properties, however, genetic modification of hMSCs, accomplished through nonviral gene delivery, could greatly advance their therapeutic potential. Furthermore, expression of multiple transgenes in hMSCs could greatly advance their clinical significance for treatment of multifaceted diseases, as individual transgenes could be expressed that target separate pathogenic drivers of complex diseases. Expressing multiple transgenes can be accomplished by delivering multiple DNA vectors encoding for each transgene, or by delivering a single poly-cistronic vector that encodes for each transgene and accomplishes expression through either use of multiple promoters, an internal ribosome entry site (IRES), or a 2A peptide sequence. These different transgene expression strategies have been used to express multiple transgenes in various mammalian cells, however, they have not been fully evaluated in difficult-to-transfect primary cells, like hMSCs. This study systematically compared four transgene expression and delivery strategies for expression of two reporter transgenes in four donors of hMSCs from two tissue sources using lipid- and polymer-mediate transfection, as follows: (i) delivery of separate DNA vectors in separate nanoparticles; (ii) delivery of separate DNA vectors combined in the same nanoparticle; (iii) delivery of a bi-cistronic DNA vector with an IRES sequence via nanoparticles; and (iv) delivery of a bi-cistronic DNA vector with a dual 2A peptide sequence via nanoparticles.

**Results:**

Our results indicate that expression of two transgenes in hMSCs, independent of expression or delivery strategy, is inefficient compared to expressing a single transgene. However, delivery of separate DNA vectors complexed in the same nanoparticle, or delivery of a bi-cistronic DNA vector with a dual 2A peptide sequence, significantly increased the number of hMSCs expressing both transgenes compared to other conditions tested.

**Conclusion:**

Separate DNA vectors delivered in the same nanoparticle and bi-cistronic DNA vectors with dual 2A peptide sequences are highly efficient at simultaneously expressing two transgenes in multiple donors of hMSCs from different tissue sources. The data presented in this work can guide the development of hMSC transfection systems for delivery of multiple transgenes, with the goal of producing clinically relevant, genetically modified hMSCs.

**Supplementary Information:**

The online version contains supplementary material available at 10.1186/s13036-023-00394-0.

## Introduction

Human mesenchymal stem cells (hMSCs) are under extensive research for applications in cell-based therapies [1–4] due to their ability to be easily isolated from numerous adult tissues [5–7], their immunomodulatory [8] and differentiation capacities [9], as well as their ability to home to sites of inflammation upon transplantation [10]. Additionally, hMSCs are immune evasive [11] and can be expanded rapidly in vitro, allowing for allogeneic transplantation of off-the-shelf cell-based therapies [12]. While the intrinsic therapeutic properties of hMSCs make them an attractive cell source for cell-based therapies, genetic modification of hMSCs through gene delivery could enhance their therapeutic properties. Nonviral gene delivery in particular, which overcomes certain limitations of viral delivery, while also presenting its own challenges of efficacy in stem cells, could enhance the innate therapeutic properties of hMSCs. For example, improving engraftment efficiency at a site of inflammation by overexpressing the pro-survival gene AKT serine/threonine kinase 1 (Akt1) [13], or endowing hMSCs with new therapeutic properties, such as expressing the full length dystrophin gene in hMSCs for Duchenne muscular dystrophy therapy [14]. Furthermore, delivery of multiple transgenes could endow hMSCs with properties that achieve more than one therapeutic effect for multifaceted disease therapies, e.g., expressing factors that mitigate the effects of both amyloid beta (Aβ) [15] and tau neurofibrillary tangles (NFTs) [16] in Alzheimer’s disease, or proteins that control both metastases and uncontrolled tumor growth in cancers [17]. However, delivery systems for efficient expression of multiple transgenes in hMSCs have not been systematically investigated.

Expression of multiple transgenes in other mammalian cells has been used to produce biopharmaceuticals and molecular drugs in Chinese hamster ovary (CHO) cells [18], as well as for biological assays in HEK293 and monkey epithelial (COS) cells [19], and for reprogramming of human somatic cells to induced pluripotent stem cells [20]. One method for expressing multiple transgenes in cells is to deliver multiple DNA vectors each encoding for a single transgene, but this method is often not as efficient at expressing multiple transgenes compared to delivering a single vector encoding for multiple transgenes, as cells can receive a heterogenous mixture of each DNA vector, leading to inequivalent expression of each transgene [18]. However, expression of multiple transgenes from a single vector requires the use of multiple promoters, which can greatly increase the overall vector size and thus potentially limit transgene expression [21]. Alternatively, separation of individual transgenes within the DNA vector by either an internal ribosome entry site (IRES) or a “self-cleaving” 2A peptide sequence [19, 22] have been used. Both IRES and 2A peptide sequences generate poly-cistronic transcripts from a single DNA vector; the IRES allows for binding of a ribosome between the transgenes for translation of downstream transgenes, whereas 2A peptide sequences cause “ribosomal skipping”, which separates each transgene as they are being translated. IRES and 2A peptide sequences have been shown to enable expression of multiple transgenes in various cell types [18, 19, 22], with a tandem 2A peptide sequence (P2A followed by T2A) generally showing higher expression of downstream transgenes compared to vectors that contain either a single 2A peptide sequence or IRES [18, 22]. However, these expression strategies have not been evaluated in clinically relevant hMSCs. Therefore, to identity DNA vector elements and delivery strategies that allow for efficient expression of multiple transgenes in hMSCs, we evaluated delivery of multiple DNA vectors, as well as bi-cistronic DNA vectors containing either an IRES or dual 2A sequence, for efficient expression of two reporter transgenes in four donors of hMSCs from two tissue sources (adipose- [hAMSCs] and bone marrow-derived [hBMSCs]) using lipid- and polymer-mediated nonviral gene delivery.

## Results

**Fig. 1:**
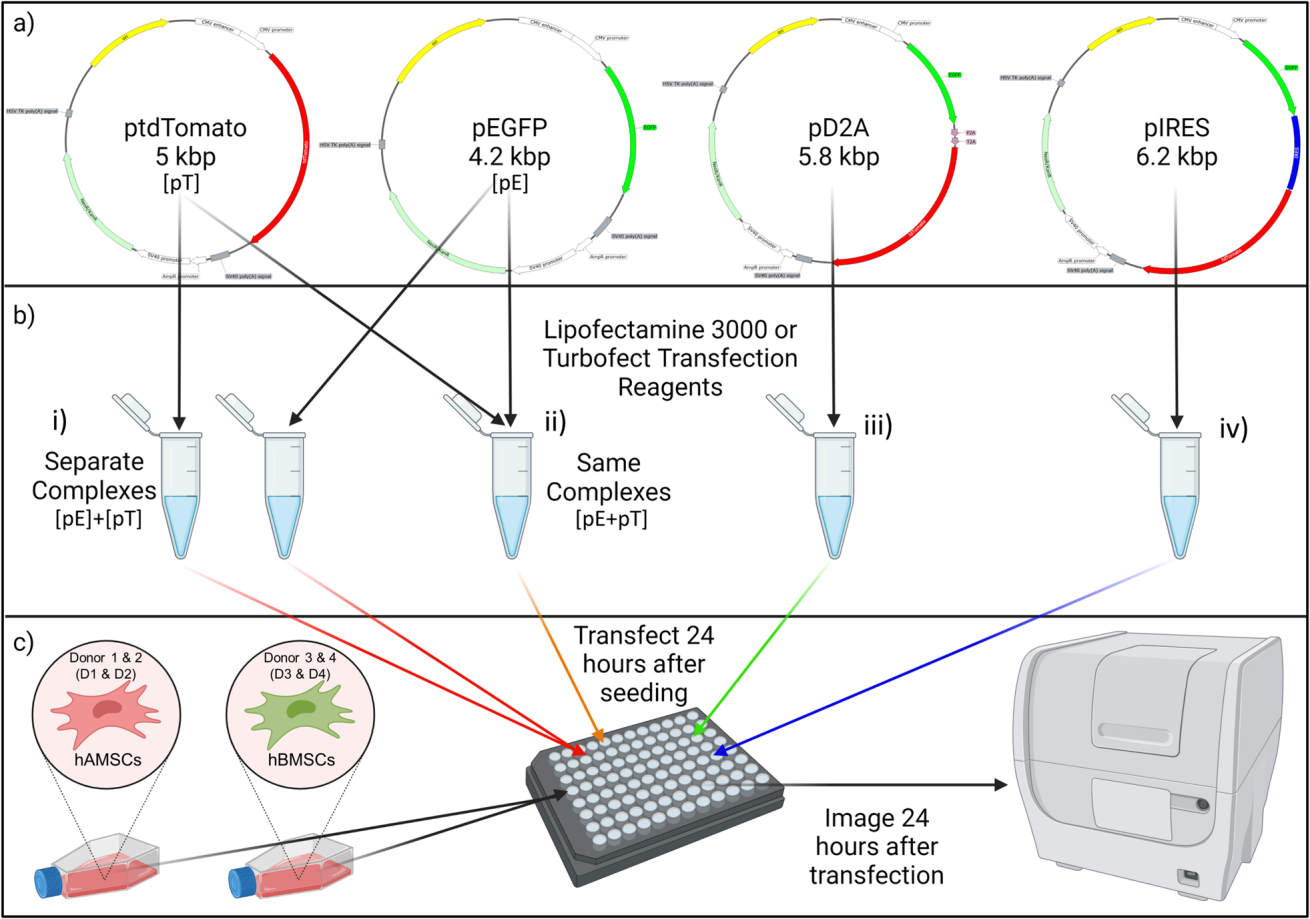
Schematic of DNA Vectors, Conditions, and Experimental Design for this Study.  **a**  Representative schematics of DNA vectors used in this study along with approximate size of DNA vectors in kilobase pairs (kbp). Sequences for each DNA vector are available at https://www.addgene.org/Angela_Pannier/. White element: CMV promoter; red element: tdTomato; green element: EGFP; grey element: SV40 polyA signal; pink element: P2A-T2A; blue element: IRES. **b**  Conditions tested for expression of multiple transgenes in hMSCs. (i) Separate complex conditions consisted of forming complexes separately for each single transgene DNA vector (pEGFP or ptdTomato, denoted as [pE]+[pT]). (ii) Same complex conditions consisted of forming complexes with both single transgene DNA vectors together (pEGFP + ptdTomato, denoted as [pE + pT]). Bi-cistronic (iii) D2A and (iv) IRES DNA vectors were formed in individual complexes. All conditions had equal mass of DNA as well as copy number of transgenes when directly compared amongst each other. Mass and copy number were equalized by addition of a promoterless pEGFP plasmid where needed. **c**  hMSCs from four donors (D1, D2, D3, & D4) and two tissue sources (adipose and bone marrow; hAMSCs and hBMSCs, respectively) were transfected with the conditions shown in « b » 24 h after seeding (4,500 and 6,000 cells/well, respectively) and imaged for transfection efficiency of each transgene 24 h after transfection

The objective of this study was to compare different transgene expression and delivery strategies for expression of two transgenes in hMSCs and investigate transfection efficiency of each transgene. Specifically, we investigated four transgene expression and delivery strategies: (i) delivery of two DNA vectors, complexed separately, each expressing a single transgene; (ii) delivery of two DNA vectors, complexed together, each expressing a single transgene; (iii) delivery of a single DNA vector expressing two transgenes separated by an internal ribosome entry site (IRES) and (iv) delivery of a single DNA vector expressing two transgenes separated by a dual 2A peptide sequence (D2A); for their ability to express two reporter transgenes (enhanced green fluorescent protein [EGFP] and tandem dimer Tomato [tdTomato]) in four donors of hMSCs from two tissue sources using the commercially available transfection reagents Lipofectamine 3000 (lipid-mediated nonviral gene delivery) or Turbofect (polymer-mediated nonviral gene delivery) for complexation (Fig. 1). The effects of each transgene expression strategy on expression of both transgenes in hMSCs were assayed by fluorescence imaging of the expressed EGFP and tdTomato transgenes, normalized by total cell count (Hoechst 33342, nuclei stain), to obtain transfection efficiencies for all conditions. It is important to note that both mass of DNA delivered, and transgene copy number (i.e., molarity of transgene), were equal when directly comparing delivery of two DNA vectors to delivery of a single DNA vector that encodes for both transgenes to appropriately compare these conditions.

### Delivery of two DNA vectors each encoding a single transgene in hMSCs

**Fig. 2:**
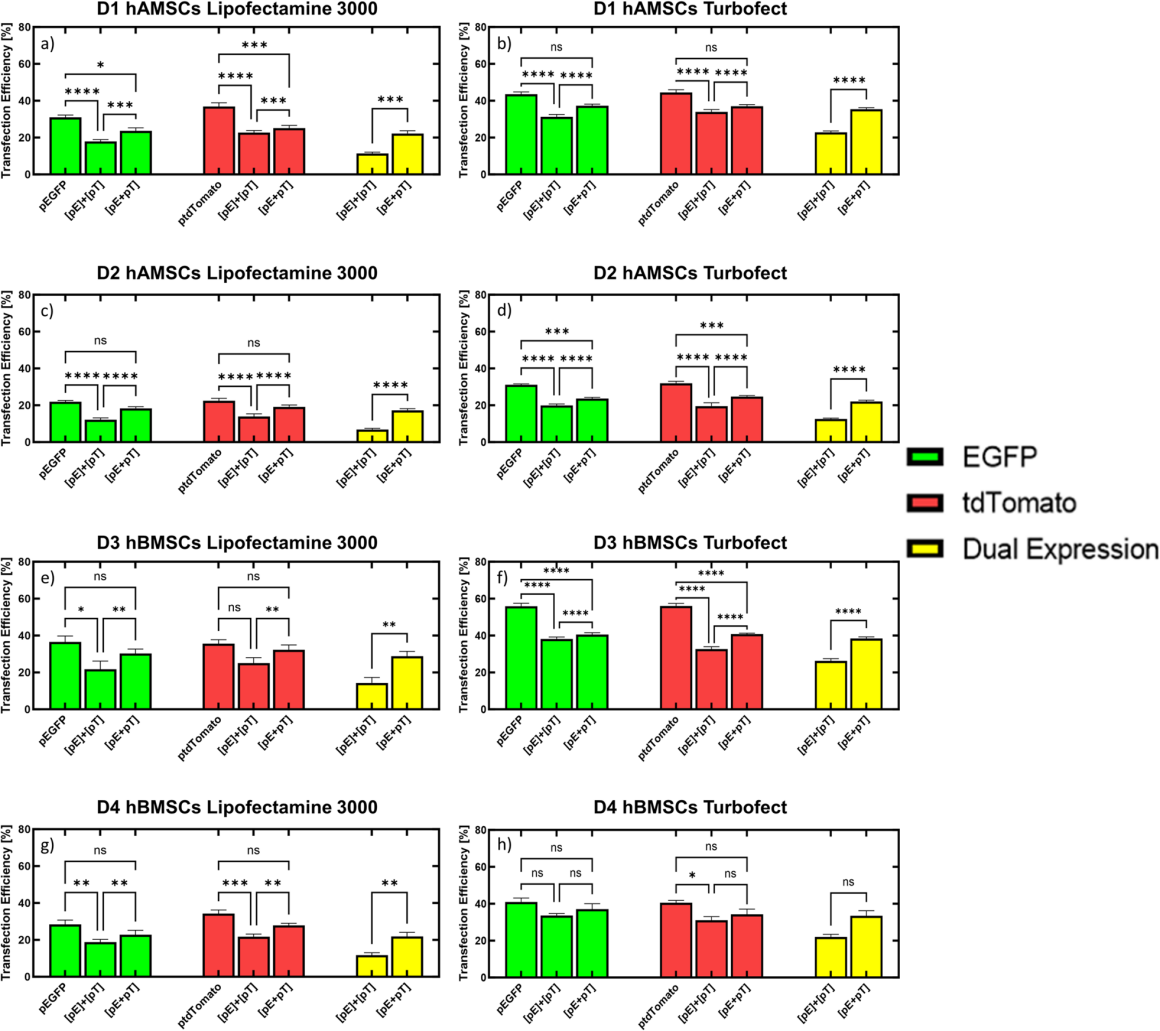
Delivery of Multiple DNA Vectors in hMSCs for Expression of Two Reporter Transgenes.  hMSCs were transfected with single transgene vectors (pEGFP or ptdTomato) complexed with Lipofectamine 3000 (**a**, **c**, **e**, & **g**) or Turbofect (**b**, **d**, **f**, & **h**) and transfection efficiencies [i.e., number of EGFP (green bars), tdTomato (red bars), and co-expressing (both EGFP and tdTomato expression, yellow bars) cells relative to total cell counts] for each transgene (EGFP or tdTomato) were compared to two transgene delivery strategies; (i) two DNA vectors delivered as separate complexes ([pE]+[pT]); and (ii) two DNA vectors delivered in the same complex ([pE + pT]). Transfection efficiencies for hMSCs expressing both EGFP and tdTomato (co-expression) was calculated by dividing the number of cells that were both EGFP and tdTomato positive by the total cell count (Hoechst, nuclear stain). **a** Transfection efficiencies for all conditions in D1 hAMSCs that used Lipofectamine 3000 as the cationic transfection reagent. **b** Transfection efficiencies for all conditions in D1 hAMSCs that made use of Turbofect as the cationic transfection reagent. **c** Transfection efficiencies for all conditions in D2 hAMSCs that used Lipofectamine 3000 as the cationic transfection reagent. **d** Transfection efficiencies for all conditions in D2 hAMSCs that made use of Turbofect as the cationic transfection reagent. **e** Transfection efficiencies for all conditions in D3 hBMSCs that used Lipofectamine 3000 as the cationic transfection reagent. **f** Transfection efficiencies for all conditions in D3 hBMSCs that made use of Turbofect as the cationic transfection reagent. **g** Transfection efficiencies for all conditions in D4 hBMSCs that used Lipofectamine 3000 as the cationic transfection reagent. **h** Transfection efficiencies for all conditions in D4 hBMSCs that made use of Turbofect as the cationic transfection reagent. All conditions have equal moles of expression cassette and mass of DNA delivered. Data represented as mean ± SEM (*n* = 6). * *p* < 0.05, ** *p* < 0.01, *** *p* < 0.001, **** *p* < 0.0001, ns, not significant (*p* > 0.05), as determined by a 2-way ANOVA with Tukey’s post hoc test

We first established baseline transfection efficiencies for each transgene following transfection with a single DNA vector. It should be noted that an expressionless plasmid (i.e., promoter removed from expression cassette of pEGFP) was added to the single DNA vector conditions to equalize both mass of pDNA and moles of each transgene delivered for the conditions tested in Fig. 2 (see *hMSC Transfection* section in Materials and Methods for more detail). For single DNA vector conditions, transfection efficiency for pEGFP was approximately 31% with Lipofectamine 3000 and 44% with Turbofect, while transfection efficiency for ptdTomato was approximately 37% with Lipofectamine 3000 and 44% with Turbofect in D1 hAMSCs (Fig. 2a and b). For D2 hAMSCs, transfection efficiency for pEGFP was approximately 22% with Lipofectamine 3000 and 31% with Turbofect, while transfection efficiency for ptdTomato was approximately 23% with Lipofectamine 3000 and 32% with Turbofect (Fig. 2c and d). For D3 hBMSCs, transfection efficiency for pEGFP was approximately 37% with Lipofectamine 3000 and 56% with Turbofect, while transfection efficiency for ptdTomato was approximately 36% with Lipofectamine 3000 and 56% with Turbofect in D3 hBMSCs (Fig. 2e and f). Lastly, for D4 hBMSCs, transfection efficiency for pEGFP was approximately 28% with Lipofectamine 3000 and 41% with Turbofect, while transfection efficiency for ptdTomato was approximately 34% with Lipofectamine 3000 and 41% with Turbofect in D4 hBMSCs (Fig. 2g and h).

Once baseline transfection levels were measured for each single transgene, we examined if delivery of two separate DNA vectors, each encoding for a single transgene (i.e., EGFP or tdTomato; Fig. 1a), would result in expression of both transgenes in hMSCs by measuring transfection efficiencies (i.e., number of EGFP, tdTomato, and EGFP + tdTomato expressing cells divided by total cell counts) resulting from delivery of the vectors in two strategies: (i) two DNA vectors delivered as separate complexes (i.e., delivery of two DNA vectors, encoding for EGFP or tdTomato, in separate cationic polymer or lipid complexes; Fig. 1bi); and (ii) two DNA vectors delivered in the same complex (i.e., delivery of two DNA vectors, encoding for EGFP or tdTomato, mixed together prior to formation in the same cationic polymer or lipid complex; Fig. 1bii). The transfection efficiencies for the delivery of two separate DNA vectors were compared to the baseline transfection efficiencies for the single transgene DNA vectors measured above.

When two DNA vectors were delivered to D1 hAMSCs in separate complexes using Lipofectamine 3000 (denoted as “[pE]+[pT]”, Fig. 1bi), transfection efficiencies for EGFP and tdTomato were significantly reduced (adjusted *p*-value < 0.0001) to 18% and 23%, respectively, compared to the single transgene conditions (Fig. 2a). Similar results were seen when Turbofect was used as the cationic carrier to separately complex and deliver the two vectors, as transfection efficiencies for both EGFP and tdTomato were significantly reduced (adjusted *p*-value < 0.0001) to 31% and 34%, respectively, compared to the single transgene conditions (Fig. 2b). When two DNA vectors were delivered to D2 hAMSCs in separate complexes using Lipofectamine 3000, transfection efficiencies for EGFP and tdTomato were significantly reduced (adjusted *p*-value < 0.0001) to 12% and 14%, respectively, compared to the single transgene conditions (Fig. 2c). Similar results were seen when Turbofect was used as the cationic carrier to separately complex and deliver the two vectors, as transfection efficiencies for EGFP and tdTomato were significantly reduced (adjusted *p*-value < 0.0001) to 20% for both transgenes compared to the single transgene conditions (Fig. 2d). When two DNA vectors were delivered to D3 hBMSCs in separate complexes using Lipofectamine 3000, transfection efficiency for EGFP was significantly reduced (adjusted *p*-value < 0.05) to 22%, however, transfection efficiency for tdTomato was not significantly reduced (adjusted *p*-value > 0.05) compared to the single transgene conditions (Fig. 2e). When Turbofect was used as the cationic carrier to separately complex and deliver the two vectors, transfection efficiencies for EGFP and tdTomato were significantly reduced (adjusted *p*-value < 0.0001) to 38% and 33%, respectively, compared to the single transgene conditions (Fig. 2f). Finally, when two DNA vectors were delivered to D4 hBMSCs in separate complexes using Lipofectamine 3000, transfection efficiencies for EGFP and tdTomato were significantly reduced (adjusted *p*-value < 0.01) to 19% and 22%, respectively, compared to the single transgene conditions (Fig. 2g). However, when Turbofect was used as the cationic carrier to separately complex and deliver the two vectors, transfection efficiency for EGFP was not significantly reduced (adjusted *p*-value > 0.05) but was significantly reduced for tdTomato (adjusted *p*-value < 0.05) compared to the single transgene conditions (Fig. 2h).

When the two DNA vectors were delivered to D1 hAMSCs by first mixing the vectors together prior to formation of the cationic complexes (denoted as “[pE + pT]”, Fig. 1bii), transfection efficiencies were reduced (adjusted *p*-value < 0.05) to 24% for EGFP and 25% for tdTomato when Lipofectamine 3000 was used as the cationic carrier (Fig. 2a), but were not significantly reduced when Turbofect was used, compared to the single transgene conditions (Fig. 2b). When two DNA vectors were delivered to D2 hAMSCs in the same complex, transfection efficiencies for EGFP and tdTomato were not significantly reduced (adjusted *p*-value > 0.05) compared to the single transgene conditions when Lipofectamine 3000 was used as the cationic carrier (Fig. 2c). However, when Turbofect was used as the cationic carrier, transfection efficiencies for EGFP and tdTomato were significantly reduced (adjusted *p*-value < 0.001) to 24% and 25%, respectively, compared to the single transgene conditions (Fig. 2d). When two DNA vectors were delivered to D3 hBMSCs in the same complex, transfection efficiencies for EGFP and tdTomato were not significantly reduced (adjusted *p*-value > 0.05) compared to the single transgene conditions when Lipofectamine 3000 was used as the cationic carrier (Fig. 2e). However, when Turbofect was used as the cationic carrier, transfection efficiencies for EGFP and tdTomato were significantly reduced (adjusted *p*-value < 0.0001) to 41% for both transgenes when compared to the single transgene conditions (Fig. 2f). When two DNA vectors were delivered to D4 hBMSCs in the same complex, transfection efficiencies for EGFP and tdTomato were not significantly reduced (adjusted *p*-value > 0.05) when either Lipofectamine 3000 or Turbofect was used as the cationic carrier compared to single transgene conditions (Fig. 2g and h). Altogether, the data suggest that expression of two transgenes is less efficient than expression of a single transgene in hMSCs, however, delivering two single transgene DNA vectors in the same complex ([pE + pT]) is more efficient at expressing the individual transgenes than delivering two single transgene DNA vectors in different complexes ([pE]+[pT]).

We next determined the percentage of hMSCs that co-expressed EGFP and tdTomato following delivery of two DNA vectors each encoding for a single transgene, either complexed in separate (Fig. 1bi) or the same complexes (Fig. 1bii), by dividing the number of EGFP positive cells that were also tdTomato positive by the Hoescht count (i.e., cell count) in each well (see *Assessment of Transfection Efficiency and Transgene Expression Levels* in Materials and Methods section for more detail). The percentage of cells that co-expressed EGFP and tdTomato when two DNA vectors were delivered in separate complexes ([pE]+[pT]) was 11% when Lipofectamine 3000 was used as the cationic carrier and 23% when Turbofect as the cationic carrier was used for D1 hAMSCs (Fig. 2a and b). The percentage of cells that co-expressed EGFP and tdTomato in D2 hAMSCs when two DNA vectors were delivered in separate complexes was 7% when Lipofectamine 3000 was used as the cationic carrier and 13% when Turbofect was used (Fig. 2c and d). The percentage of cells that co-expressed EGFP and tdTomato in D3 hBMSCs when two DNA vectors were delivered in separate complexes was 14% when Lipofectamine 3000 was used as the cationic carrier and 26% when Turbofect was used (Fig. 2e and f). Lastly, the percentage of cells that co-expressed EGFP and tdTomato when two DNA vectors were delivered in separate complexes was 12% in D4 hBMSCs when Lipofectamine 3000 was used as the cationic carrier and 22% when Turbofect was used (Fig. 2g and h).

Finally, comparing the percentage of cells co-expressing EGFP and tdTomato when the two DNA vectors were delivered in the same complex to delivery of two DNA vectors in separate complexes showed a significant increase (adjusted *p*-value < 0.01) in co-expression transfection efficiencies regardless of the cationic carrier used or the donor of hMSC delivered to (Fig. 2a-g), except for D4 hBMSCs when Turbofect was used as the cationic carrier (Fig. 2h). However, the percentage of cells co-expressing EGFP and tdTomato in D4 hBMSCs when two DNA vectors were delivered in the same complex was still higher (34%) than delivery of two DNA vectors in separate complexes (22%; Fig. 2h), demonstrating that in all conditions studied with single transgene vectors, inclusion of both vectors within the same complex resulted in the highest co-expression transfection efficiencies.

### Delivery of a single, bi-cistronic Vector in hMSCs

**Fig. 3:**
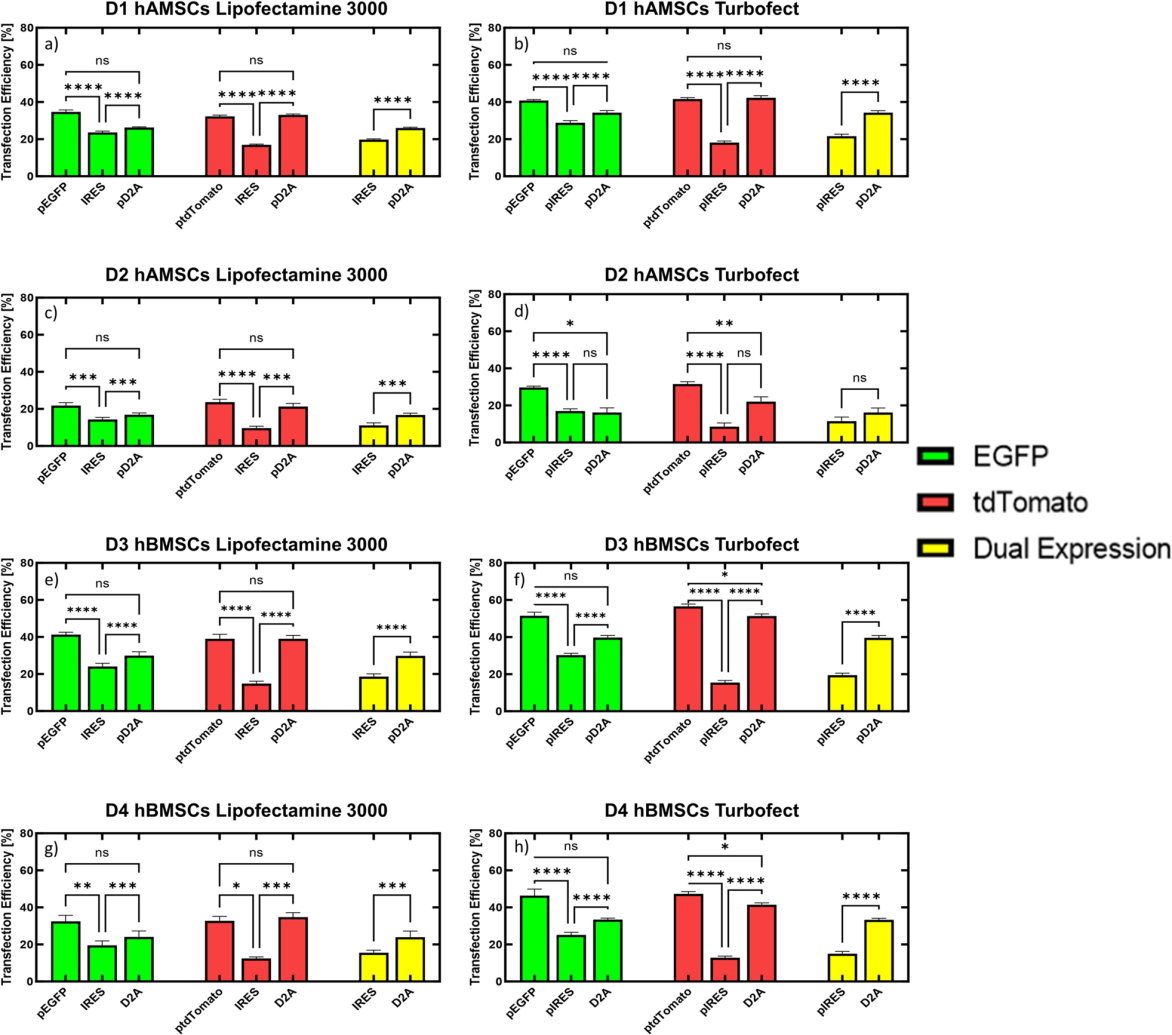
Delivery of Bi-Cistronic DNA Vectors with an IRES or D2A Sequence for Expression of Two Reporter Transgenes in hMSCs.  hMSCs were transfected with bi-cistronic DNA vectors complexed with Lipofectamine 3000 (**a**, **c**, **e**, & **g**) or Turbofect (**b**, **d**, **f**, & **h**) and transfection efficiencies for each transgene (EGFP, green bars, or tdTomato, red bars) were compared to the expression levels of single transgene vectors (pEGFP or ptdTomato) in D1 hAMSCs (**a** & **b**), D2 hAMSCs (**c** & **d**), D3 hBMSCs (**e** & **f**), and D4 hBMSCs (**g** & **h**). Transfection efficiencies for hMSCs expressing both EGFP and tdTomato (co-expression, yellow bars) was calculated by dividing the number of cells that were both EGFP and tdTomato positive by the total cell count (Hoechst, nuclear stain). All conditions have equal moles of expression cassette and mass of DNA delivered. Data represented as mean ± SEM (*n* = 6). * *p* < 0.05, **, *p* < 0.01, *** *p* < 0.001, **** *p* < 0.0001. ns, not significant (*p* > 0.05), as determined by a 2-way ANOVA with Tukey’s post hoc test

Next, we investigated whether delivering two transgenes on the same DNA vector, separated by an IRES or D2A sequence, could efficiently co-express both transgenes in hMSCs. Transfection efficiencies (i.e., number of EGFP, tdTomato, and co-expressing cells divided by total cell counts) were measured for bi-cistronic IRES and D2A DNA vector conditions and compared to single DNA vector conditions (i.e., delivery of a single DNA vector encoding either EGFP or tdTomato). It should be noted that in these studies, no expressionless plasmid was added to these conditions tested since a given mass of pDNA has similar moles of expression cassette, therefore, single transgene DNA vector transfection efficiencies were again measured to obtain a baseline using DNA doses that matched those of the bi-cistronic vectors (i.e., same mass of pDNA and moles of expression cassette). At this dose, single DNA vector transfection efficiencies for D1 hAMSCs were 35% and 32% for EGFP and tdTomato, respectively, when Lipofectamine 3000 was used as the cationic carrier, and 41% and 42%, respectively, when Turbofect was used as the cationic carrier (Fig. 3a and b). However, when the bi-cistronic IRES DNA vector was used at the same dose as the single transgene vectors, transfection efficiencies for EGFP and tdTomato were significantly reduced (adjusted *p*-value < 0.0001) to 29% and 18%, respectively, compared to the transfection efficiencies for the single transgene DNA vector conditions, but were not significantly reduced (adjusted *p*-value > 0.05) for the bi-cistronic D2A DNA vector, regardless of cationic carrier used (Fig. 3a and b). Furthermore, the bi-cistronic D2A DNA vector produced significantly higher transfection efficiencies (adjusted *p*-value < 0.0001) for both transgenes compared to the bi-cistronic IRES DNA vector, regardless of cationic carrier used (Fig. 3a and b). Single DNA vector transfection efficiencies for D2 hAMSCs were 22% and 24% for EGFP and tdTomato, respectively, when Lipofectamine 3000 was used as the cationic carrier, and 30% and 32%, respectively, when Turbofect was used as the cationic carrier (Fig. 3c and d). Transfection efficiencies for both EGFP and tdTomato were significantly reduced (adjusted *p*-value < 0.001) to 14% and 10% for the bi-cistronic IRES DNA vector compared to the transfection efficiencies for the single transgene DNA vector conditions, regardless of cationic carrier used (Fig. 3c and d). Transfection efficiencies for EGFP and tdTomato in D2 hAMSCs were not significantly reduced (adjusted *p*-value > 0.05) for the bi-cistronic D2A DNA vector compared to the single transgene conditions when Lipofectamine 3000 was used as the cationic carrier (Fig. 3c), but were significantly reduced (adjusted *p*-value < 0.05) to 16% and 22%, respectively, compared to the single transgene conditions when Turbofect was used as the cationic carrier (Fig. 3d). Conversely, the bi-cistronic D2A DNA vector produced significantly higher transfection efficiencies (adjusted *p*-value < 0.001) for EGFP and tdTomato compared to the bi-cistronic IRES DNA vector when Lipofectamine 3000 was used as the cationic carrier (Fig. 3c), while no significant difference in transfection efficiency for each transgene was seen between the bi-cistronic IRES and D2A DNA vectors when Turbofect was used as the cationic carrier (Fig. 3d).

Single DNA vector transfection efficiencies for D3 hBMSCs were 41% and 39% for EGFP and tdTomato, respectively, when Lipofectamine 3000 was used as the cationic carrier (Fig. 3e), and 52% and 57%, respectively, when Turbofect was used as the cationic carrier (Fig. 3f). Transfection efficiencies for EGFP and tdTomato were significantly reduced (adjusted *p*-value < 0.0001) for the bi-cistronic IRES DNA vector compared to the transfection efficiencies for the single transgene DNA vector conditions, regardless of complexing reagent used, while the bi-cistronic D2A DNA vector only had a significant reduction in tdTomato transfection efficiency (adjusted *p*-value < 0.05) compared to the single transgene condition when Turbofect was used as the cationic carrier (Fig. 3e and f). Moreover, the bi-cistronic D2A DNA vector produced significantly higher transfection efficiencies (adjusted *p*-value < 0.0001) for EGFP and tdTomato compared to the bi-cistronic IRES DNA vector regardless of cationic carrier used (Fig. 3e and f). Lastly, single DNA vector transfection efficiencies for D4 hBMSCs were 33% and 33% for EGFP and tdTomato, respectively, when Lipofectamine 3000 was used as the cationic carrier, and 46% and 47%, respectively, when Turbofect was used as the cationic carrier (Fig. 3g and h). Transfection efficiencies for EGFP and tdTomato were significantly reduced (adjusted *p*-value < 0.05) for the bi-cistronic IRES DNA vector compared to the transfection efficiencies for the single transgene DNA vector conditions, regardless of cationic carrier used (Fig. 3g and h). Conversely, the transfection efficiencies for EGFP and tdTomato were not significantly reduced (adjusted *p*-value > 0.05) for the bi-cistrionic D2A DNA vector compared to the single transgene conditions, except for tdTomato when Turbofect was used as the cationic carrier (Fig. 3h). Furthermore, the bi-cistronic D2A DNA vector produced significantly higher transfection efficiencies (adjusted *p*-value < 0.001) for EGFP and tdTomato compared to the bi-cistronic IRES DNA vector regardless of cationic carrier used (Fig. 3g and h). Altogether, the data suggest that expressing two transgenes from a single, bi-cistronic DNA vector is less efficient than expressing the same transgene from a mono-cistronic DNA vector in hMSCs. Moreover, inclusion of a D2A peptide sequence between two distinct transgenes encoded on a single DNA vector can significantly increase expression of both transgenes compared to inclusion of an IRES between those transgenes.

Finally, when comparing the percentage of cells co-expressing both transgenes (i.e., number cells that express EGFP and tdTomato divided by total cell count) for the two bi-cistronic DNA vectors (Fig. 3a-h), the D2A DNA vector produced a significantly higher percentage of cells (adjusted *p*-value < 0.001) that expressed both EGFP and tdTomato compared to the IRES DNA vector in all donors regardless of cationic carrier used, except for D2 hAMSCs when Turbofect was used as the cationic carrier (Fig. 3d), suggesting that in all conditions studied with bi-cistronic vectors, inclusion of D2A peptide sequence resulted in the highest co-expression transfection efficiencies.

### Identification of transfection strategies for simultaneous co-expression of two transgenes in hMSCs

**Fig. 4:**
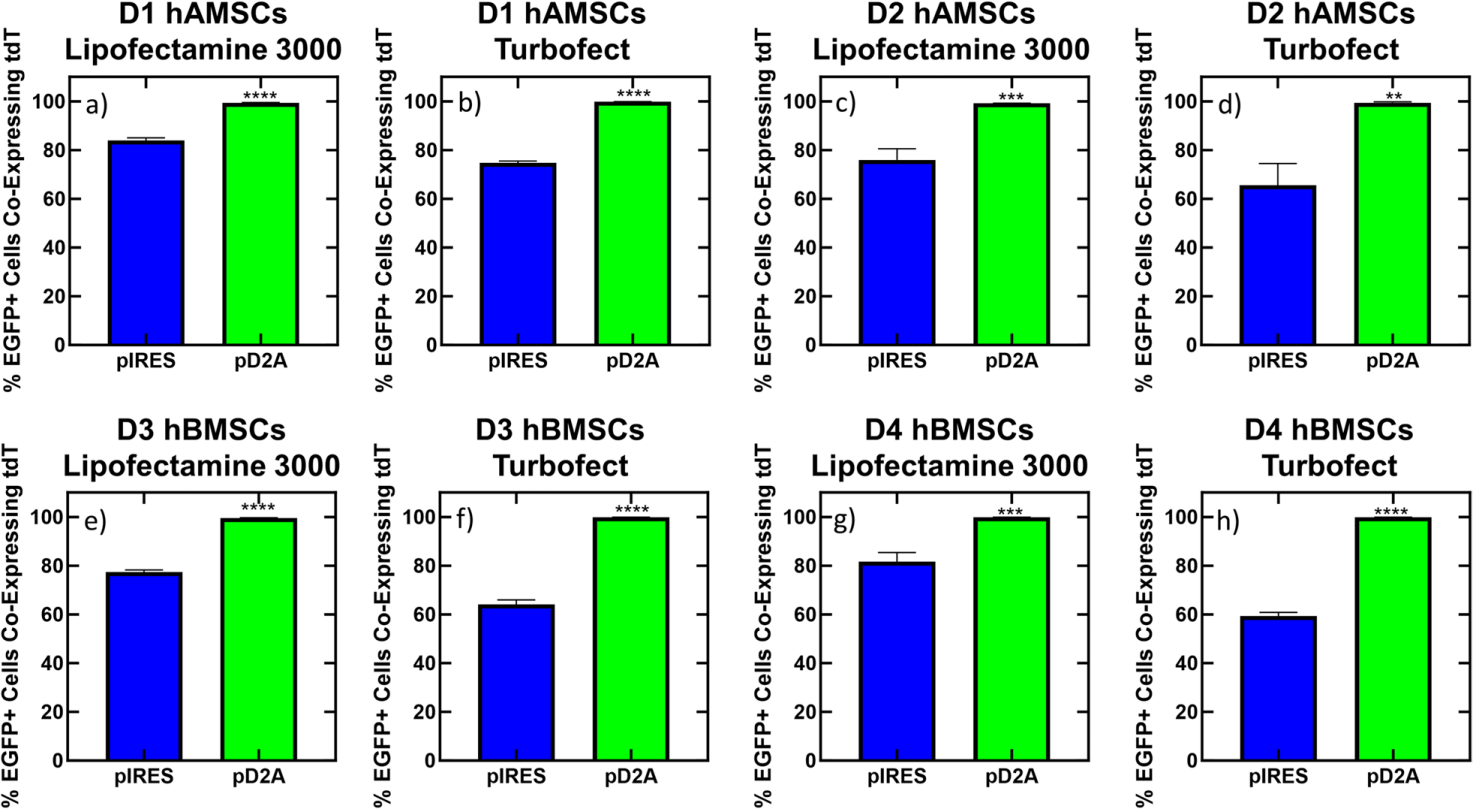
Percent of Transfected hMSCs Simultaneously Expressing Both Transgenes from Bi-Cistronic DNA Vectors. hMSCs were transfected with either the bi-cistronic pD2A or pIRES DNA vector complexed with Lipofectamine 3000 (**a**, **c**, **e**, & **g**) or Turbofect (**b**, **d**, **f**, & **h**) and assayed for transgene expression 24 h after transfection for D1 hAMSCs (**a** & **b**), D2 hAMSCs (**c** & **d**), D3 hBMSCs (**e** & **f**), and D4 hBMSCs (**g** & **h**). The percent of EGFP expressing cells that are expressing tdTomato was calculated by dividing the number of EGFP positive cells that were also tdTomato positive by the number of EGFP positive cells. The reasoning behind normalizing to EGFP positive cells, as well as counting EGFP positive cells that are also tdTomato positive, as opposed to normalizing to tdTomato positive cells or tdTomato positive cells that are also EGFP positive, is that translation of the upstream transgene (EGFP) is required for D2A sequences before translation of the downstream transgene can occur. Data represented as mean ± SEM (*n* = 6). * indicates significance relative to pIRES. *** *p* < 0.001, **** *p* < 0.0001, as determined by a two-tailed T-test

Next, we calculated the percentage of cells that were successfully transfected (i.e., expressing EGFP) that were simultaneously expressing both transgenes from the bi-cistronic IRES and D2A DNA vector conditions by dividing the number of EGFP positive cells that were also tdTomato positive by the number of EGFP positive cells (Fig. 4). The reasoning behind normalizing to EGFP positive cells, as well as counting EGFP positive cells that are also tdTomato positive, as opposed to normalizing to tdTomato positive cells or counting tdTomato positive cells that are also EGFP positive, is that for D2A sequences, translation of the upstream transgene (EGFP) is required before translation of the downstream transgene (tdTomato) can occur. The bi-cistonic D2A DNA vector produced significantly more successfully transfected cells (adjusted *p*-value < 0.01) that were co-expressing EGFP and tdTomato compared to the bi-cistronic IRES DNA vector regardless of hMSC donor, tissue source, or cationic carrier used (Fig. 4). The bi-cistronic IRES DNA vector resulted in 60–84% of successfully EGFP transfected cells simultaneously co-expressing tdTomato, whereas the bi-cistronic D2A DNA vector resulted in 99-100% of successfully EGFP transfected cells simultaneously co-expressing tdTomato, regardless of hMSC donor, tissue source, or cationic carrier used (Fig. 4, Supplemental Fig. 1), suggesting that in all conditions studied with bi-cistronic vectors, inclusion of D2A peptide sequence resulted in the highest efficiency of expressing the downstream transgene (tdTomato) in cells that were expressing the upstream transgene (EGFP).

**Fig. 5:**
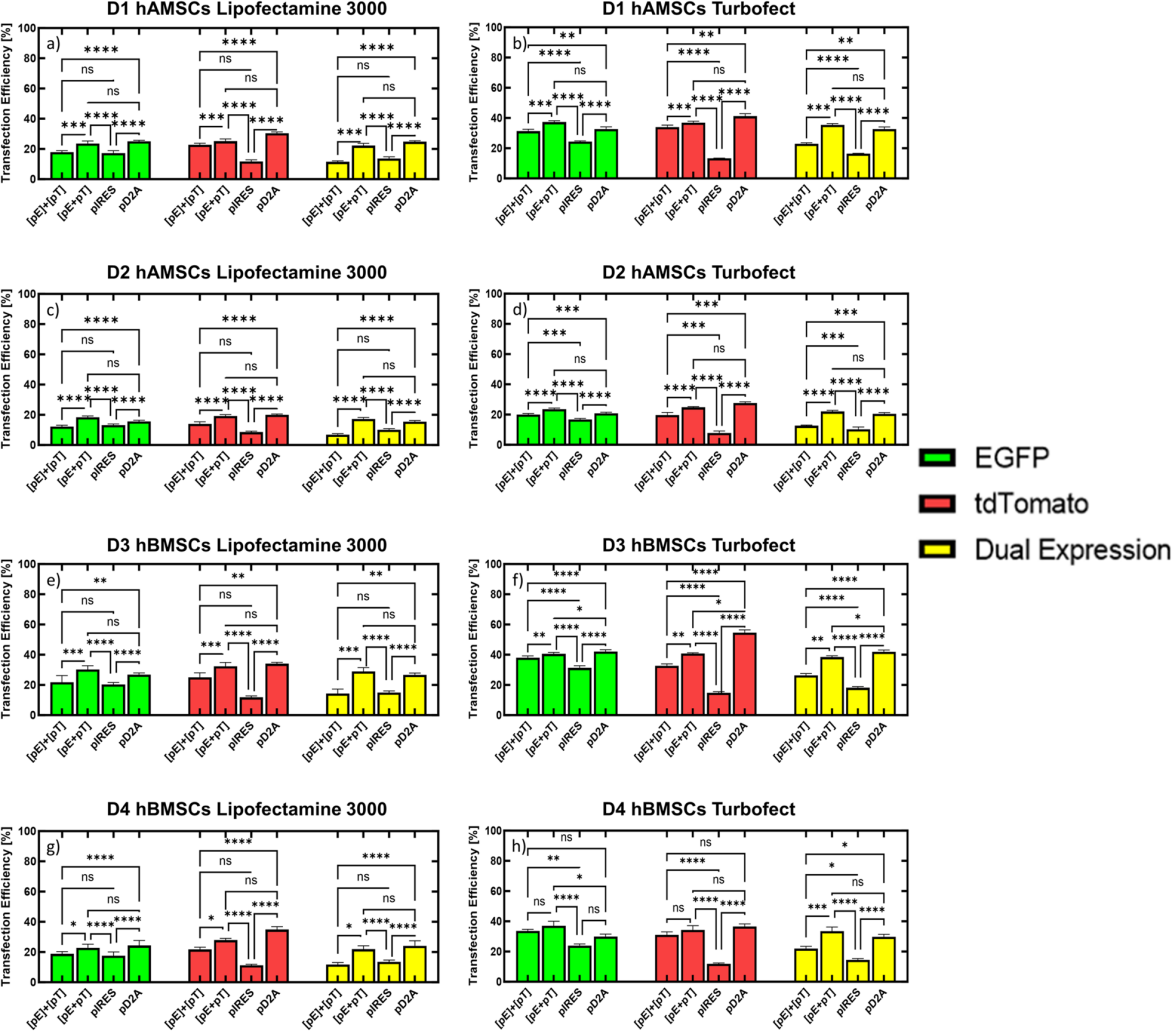
Equal Mass of DNA and Copy Number of Transgenes Comparison for Delivery of Two DNA Vectors to Delivery of Bi-Cistronic DNA Vectors for Expression of Two Transgenes in hMSCs.  hMSCs were transfected with single transgene DNA vectors delivered in separate complexes ([pE]+[pT]) the same complex ([pE + pT]) or bi-cistronic DNA vectors (pD2A or pIRES) with an equal mass of DNA and copy number of transgenes delivered. All four conditions were complexed with Lipofectamine 3000 (**a**, **c**, **e**, & **f**) or Turbofect (**b**, **d**, **f**, & **h**). Transgene expression for each condition was assayed 24 h after transfection for D1 hAMSCs (**a** & **b**), D2 hAMSCs (**c** & **d**), D3 hBMSCs (**e** & **f**), and D4 hBMSCs (**g** & **h**). Expression of both transgenes simultaneously in hMSCs (co-expression) was calculated by dividing the number of cells that were both EGFP and tdTomato positive by the total cell count (Hoechst, nuclear stain). Data represented as mean ± SEM (*n* = 6). * *p* < 0.05, ** *p* < 0.01, *** *p* < 0.001, **** *p* < 0.0001. ns, not significant (*p* > 0.05), as determined by a 2-way ANOVA with Tukey’s post hoc test

We next directly compared delivery strategies for expressing two transgenes (i.e., [pE]+[pT], [pE + pT], or delivery of bi-cistronic DNA vectors that express two transgenes via an IRES or D2A sequence; Fig. 5). To properly compare these conditions, addition of expressionless vector to the bi-cistronic DNA vector conditions was needed to equalize mass of pDNA and molarity of each transgene for each condition. Directly comparing delivery of two DNA vectors in separate or the same complex, to delivery of a single bi-cistronic DNA vector with an IRES or D2A with both mass of DNA and molarity of expression cassette normalized across all conditions, showed that inclusion of an IRES sequence resulted in significantly reduced (adjusted *p*-value < 0.05) expression of tdTomato compared to all other conditions (Fig. 5a-h). However, the inclusion of an IRES sequence did not significantly reduce tdTomato expression compared to delivery of two DNA vectors in separate complexes, for all donors using Lipofectamine 3000 as the cationic carrier (adjusted *p*-value > 0.05). Furthermore, the bi-cistronic D2A DNA vector led to significantly higher transfection efficiencies (adjusted *p*-value < 0.05) of cells co-expressing EGFP and tdTomato compared to all other conditions, except when compared to delivery of two transgenes in the same complex. However, the bi-cistronic D2A DNA vector showed significantly higher co-expression compared to delivery of two transgene in the same complex in D3 hBMSCs when Turbofect was the used as the cationic carrier (Fig. 5f). These results suggests that in all conditions studied, delivery of a single DNA vector with a D2A peptide sequence, as well as delivery of two DNA vectors in the same complex, resulted in the highest co-expression transfection efficiencies.

**Fig. 6:**
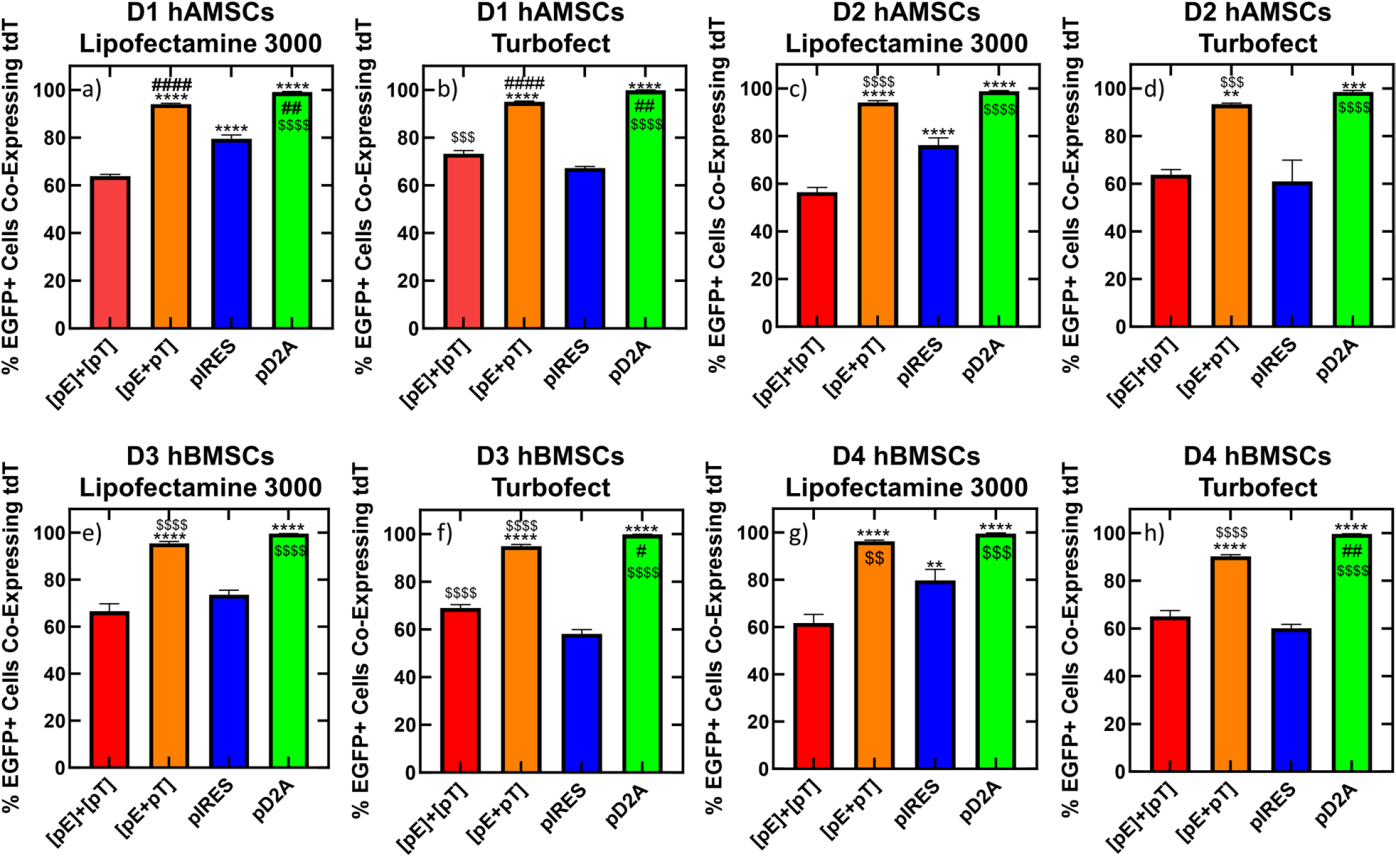
Comparison of Percent of Transfected hMSCs Simultaneously Expressing Both Transgenes from Delivery of Two DNA Vectors to Delivery of Bi-Cistronic DNA Vectors when Equal Mass of DNA and Copy Number of Transgenes are Delivered.  hMSCs were transfected with single transgene DNA vectors delivered in separate complexes ([pE]+[pT]), the same complex ([pE + pT]) or bi-cistronic DNA vectors (pD2A or pIRES) with an equal mass of DNA and copy number of transgenes delivered. All four conditions were complexed with Lipofectamine 3000 (**a**, **c**, **e**, & **g**) or Turbofect (**b**, **d**, **f**, & **h**). Transgene expression for each condition was assayed 24 h after transfection for D1 hAMSCs (**a** & **b**), D2 hAMSCs (**c** & **d**), D3 hBMSCs (**e** & **f**), and D4 hBMSCs (**g** & **h**). The percent of EGFP expressing cells that are expressing tdTomato was calculated by dividing the number of EGFP positive cells that were also tdTomato positive by the number of EGFP positive cells. The reasoning behind normalizing to EGFP positive cells, as well as counting EGFP positive cells that are also tdTomato positive, as opposed to normalizing to tdTomato positive cells or tdTomato positive cells that are also EGFP positive, is that translation of the upstream transgene (EGFP) is required for D2A sequences before translation of the downstream transgene can occur. Data represented as mean ± SEM (*n* = 6). * indicates significance relative to [pE]+[pT]. *** *p* < 0.001, **** *p* < 0.0001. # indicates significance relative to [pE+pT]. # *p* < 0.05, ## *p* < 0.01. $ indicates significance relative to pIRES. $ $ *p* < 0.01, $ $ $ *p* < 0.001, $ $ $ $ *p* < 0.0001, as determined by a 2-way ANOVA with Tukey’s post hoc test. Only significant comparisons are shown for clarity

Finally, we calculated the percentage of EGFP-expressing cells that were simultaneously expressing Tdtomato for delivery of two DNA vectors in separate or the same complex, as well as for the bi-cistronic IRES and D2A DNA vectors, with mass of DNA and moles of each transgene delivered equalized across all conditions. Delivery of two DNA vectors in separate complexes led to 56–73% of successfully EGFP-transfected cells also expressing tdTomato, across all donors and cationic carries tested (Fig. 6). Similarly, the bi-cistronic IRES DNA vector led to 58–80% of successfully EGFP-transfected cells simultaneously expressing tdTomato across all donors and cationic carries tested (Fig. 6). Conversely, delivery of two DNA vectors in the same complex led to 90–96% of successfully EGFP-transfected cells simultaneously expressing Tdtomato across all donors and cationic carriers tested, which was significantly higher (adjusted *p*-value < 0.01) than delivery of two DNA vectors in separate complexes and the bi-cistronic IRES DNA vector for all donors and cationic carriers tested (Fig. 6). However, the bi-cistronic D2A DNA vector led to 99–100% of successfully EGFP-expressing cells simultaneously expressing Tdtomato across all donors and cationic carriers tested, which was significantly higher (adjusted *p*-value < 0.05) than all tested conditions except for delivery of two DNA vectors in the same complex using Lipofectamine 3000 as the cationic carrier in D2, D3, and D4 hMSCs and when using Turbofect as the cationic carrier in D2 hAMSCs (Fig. 6). Altogether, these results suggest that a bi-cistronic DNA vector with a D2A peptide sequence can mediate up to 100% of successfully transfected hMSCs (i.e., cells expressing the first transgene) simultaneously expressing the second transgene, and delivery of two DNA vectors in the same complex can mediate up to 96% of successfully transfected hMSCs simultaneously expressing the second transgene in multiple donors, regardless of cationic carrier.

## Discussion

hMSCs are primary cells under intense research for cell-based therapies [1–4] due to their unique properties [5–8, 10–12], which can be enhanced through genetic modification. Viral gene delivery can be efficient [23, 24], but poses significant safety and production issues [25, 26], while nonviral gene delivery, which can overcome the safety and production issues associated with viral gene delivery [25, 27], is inefficient [28–34], especially in hMSCs. Delivery of multiple transgenes to hMSCs could greatly enhance their therapeutic value by tailoring the cells for therapies targeting multifaceted diseases or for use in reprogramming, differentiation, or genome editing strategies, however, nonviral delivery systems to express multiple transgenes have not been evaluated in hMSCs. Therefore, the goal of this work was to compare four expression and delivery strategies for expression of two transgenes (i.e., delivery of two single transgene DNA vectors complexed separately or together, as well as delivery of a single DNA vector that can express two transgenes through the use of an IRES or D2A peptide sequence, see Fig. 1) in hMSCs from four donors and two tissue sources using two commercially available transfection regents.

We first examined if delivery of two DNA vectors, each encoding for a single transgene (either EGFP or tdTomato), would result in expression of both transgenes in hMSCs (Fig. 2) by delivering two DNA vectors, either in separate complexes or the same complex (Fig. 1) and comparing the transfection efficiencies for each transgene to the transfection efficiency of the single transgene when delivered alone, as well as determining the transfection efficiency for hMSCs that co-expressed both transgenes. Delivery of two separate DNA vectors, each encoding a single transgene in separate complexes, was not an efficient way to express two transgenes in hMSCs, as the transfection efficiencies for each transgene were lower when they were delivered together than transfection efficiencies when a single DNA vector encoding for one of the transgenes was delivered (Fig. 2). In previous studies, Bayat and colleagues observed the lowest titers of antibodies in CHO cells when the transgenes needed for antibody production were delivered on two separate vectors [18]. The authors hypothesized that the low antibody expression levels from delivery of two DNA vectors was possibly due to inequivalent delivery of each DNA vector into the transfected cells [18]. However, we showed that delivery of two separate DNA vectors each encoding a single transgene complexed together resulted in up to 96% of successfully transfected cells (EGFP+) co-expressing both transgenes (Fig. 6), presumably because both vectors are incorporated into the same complex, which increases the chance any transfected cell has internalized both vectors, as opposed to delivering separate complexes. It should be noted though that Bayat and colleagues did not make it clear whether the vectors were mixed prior to complexation or not, therefore making it difficult to compare results between that study and our results presented here. Furthermore, a more drastic reduction in transfection outcomes from delivery of multiple DNA vectors has been observed in human induced pluripotent stem cell (iPSCs) production. For example, Yu and colleagues delivered three episomal DNA vectors to human foreskin fibroblasts, each expressing a combination of six transgenes (Oct4, Sox2, Nanog, Klf4, c-Myc, or Lin28), and achieved successful reprogramming of only three to six iPSC colonies per million input cells (0.0003–0.0006%) [35]. However, Montserrat and colleagues were able to generate 61 iPSC colonies from 400,000 human foreskin fibroblast input cells (0.015%) by delivering a poly-cistronic DNA vector that expressed the four reprogramming transgenes (Oct4, Sox2, Klf4, and c-Myc) using 2A peptide sequences [36, 37], suggesting that poly-cistronic DNA vectors may enhance expression of multiple transgenes in cells.

Indeed, polycistronic DNA vectors have shown efficient expression of multiple transgenes in clinically relevant cells, such as human embryonic stem cells (hESCs) [38], as well as hMSCs, as we observed for the first time in this report (Figs. 3, 4, 5 and 6). Here, we cloned and tested two bi-cistronic DNA vectors, which made use of either an IRES or D2A peptide sequence, to express two transgenes from a single DNA vector in hMSCs. We showed that the D2A peptide sequence significantly increased the percentage of successfully transfected (EGFP+) hMSCs that simultaneously expressed both transgenes compared to an IRES sequence or delivery of two DNA vectors, regardless of complexing reagent or hMSC donor or tissue source, except for D2 hAMSCs, or D3 and D4 hBMSCs when Lipofectamine 3000 was used as the cationic carrier (Fig. 6). Others have reported similar results in other cells, for instance, Hasegawa and colleagues observed higher co-expression of an EGFP and DsRed reporter transgene in hESCs when a single 2A peptide sequence separated the two transgenes compared to an IRES [38]. In addition, we also observed less tdTomato expression (i.e., the transgene downstream of EGFP) from the bi-cistronic IRES DNA vector compared to the bi-cistronic D2A DNA vector across all hMSC donors and cationic carriers tested. Similarly, Fang and colleagues observed higher levels of expression of a rat anti-mouse vascular endothelial cell growth factor receptor 2 (VEGFR2) immunoglobin G1 (IgG1) monoclonal antibody in HEK293 cells when the antibody heavy and light chains were separated by a single 2A peptide sequence compared to an IRES [39]. Finally, EGFP expression from the bi-cistronic IRES DNA vector was significantly lower than EGFP expression from the bi-cistronic D2A DNA vector (Fig. 3), which was unexpected as both transcripts should be translated in a cap-dependent manner. One potentical explanation for the lower EGFP expression from the bi-cistronic IRES DNA vector compared to the bi-cistronic D2A DNA vector could be due to differences in mRNA secondary structures, which can greatly affect transgene expression [40, 41]. IRES elements are known to have secondary structures that facilitate ribosomal binding [42], which may interfere with the mRNA structure of the whole bi-cistronic transcript. This potential mRNA secondary structure in transcripts from the bi-cistronic IRES DNA vector might also explain the overall lower transfection efficiencies observed in this report for each transgene when IRES was used in the DNA vector, as all other elements encoded in each DNA vector were identical (i.e., promoter, transgenes, and poly adenylation signal).

While the D2A bi-cistronic DNA vector resulted in robust expression of both transgenes in hMSCs in this work, the lower expression levels of the transgene downstream of an IRES compared to the upstream transgene could be useful for controlling the expression of each transgene. For instance, Schlatter and colleagues showed that the optimal ratio of heavy chain to light chain for recombinant monoclonal antibody production in CHO cells were different between transient and stable expression systems, with transient systems having an optimal ratio of 3:2 for heavy to light chain [43]. Furthermore, this ratio differed from the perceived optimal ratio of 1:1 for heavy to light chains, as monoclonal antibodies naturally have equimolar ratios of heavy and light chains, indicating that an IRES based transient transfection system could be used to fine tune the ratio of transgenes for therapeutic applications.

The improvement in dual transgene expression with the 2A peptide sequence compared to the IRES may involve the mechanisms by which the downstream transgenes are translated. For a transgene downstream of an IRES to be translated, a ribosome must bind to the IRES in a cap-independent manner [44], which has been shown to be in-efficient compared to cap-dependent translation [45]. Conversely, the 2A peptide sequence only requires ribosomal binding to the first transgene mRNA (cap-dependent translation) as the 2A peptide sequence is entirely transcribed and translated along with all downstream transgenes [46]. The transgenes are then hydrolytically cleaved between the glycine and proline amino acids within the 2A peptide sequence [46], thereby creating individual poly-peptide sequences for each transgene in an equimolar fashion. Therefore, the lower downstream transgene expression and co-expression levels for IRES compared to D2A could be due to the in-efficient, cap-independent translation from the IRES. However, Kim and colleagues reported that a single 2A peptide sequence, while having a cleavage efficiency of close to 100%, still results in some uncleaved protein due to incomplete ‘ribosome skipping’, as determined using a Western blot [47]. To address the issue of inefficient cleavage with a single 2A peptide sequence, Pan and colleagues connected the porcine Teschovirus-1 2A (P2A) and the Thosea asigna virus 2A (T2A) to create a D2A that led to undetectable levels of unseparated proteins [22], presumably through more efficient ‘ribosomal skipping’. Indeed, we also observed lower transgene expression of downstream transgenes when single 2A peptide sequences were used (unpublished data) compared to when a D2A was used as reported in this study, further supporting that a D2A peptide sequence is highly efficient at expressing downstream transgenes.

Finally, our previous reports have shown a great deal of variability in transfection success related to hMSC donor, tissue source, and transfection reagent used [29–33, 48]. These variables were tested in this study, and individual transfection efficiencies did differ significantly amongst the various conditions tested in this study. For example, D3 hBMSCs had overall higher transfection efficiencies compared to D4 hBMSCs and both donors of hAMSCs for all conditions tested. We have noted higher transfection efficiency in hBMSCs relative to hAMSCs in our previous work [48], which was potentially attributed to differences in proliferation rates and transfection-induced cytotoxicity with different tissue sources of the cells [48]. Additionally, we have also noted differences in transfection efficiencies between donors of hMSCs in our previous studies [29, 30, 32, 33, 48]. However, the number of donors and tissue sources tested in this current study are not large enough to enable correlation between transfection outcomes and hMSC attributes. Furthermore, the overall transfection trends for the DNA vectors and delivery methods evaluated were similar across all donors, tissue sources, and transfection reagents tested. These findings suggests that vector design effects, as they pertain to expression of multiple transgenes in hMSCs, do not meaningfully differ between different donors, tissue sources, or transfection reagents. Therefore, rational vector design can broadly advance hMSC transfection systems.

## Conclusions

This work systematically compared four transgene delivery and expression strategies: (i) delivery of two DNA vectors, complexed separately, each expressing a single transgene; (ii) delivery of two DNA vectors, complexed together, each expressing a single transgene; (iii) delivery of a bi-cistronic DNA vector expressing two transgenes separated by an IRES; and (iv) delivery of a bi-cistronic DNA vector expressing two transgenes separated by a D2A) for efficient co-expression of two reporter transgenes (EGFP and tdTomato) in four donors of hMSCs from two tissue sources using the commercially available Lipofectamine 3000 and Turbofect transfection reagents. Analyzing transfection efficiencies of both transgenes for the four delivery systems showed that the bi-cistronic DNA vector with a D2A sequence produced the most co-expressing hMSCs compared to the other delivery systems, with 99–100% of successfully transfected (i.e., expressing a transgene) hMSCs simultaneously expressing both transgenes. Alternatively, delivery of two single transgene DNA vectors complexed together resulted in up to 96% of successfully transfected cells expressing both transgenes. Furthermore, our results indicate that an IRES sequence could be a strategy to control the expression levels of downstream transgenes, which could be useful in therapeutic applications that require one transgene to be expressed at higher levels. Lastly, the transgene expression strategies outlined in this study had similar trends across hMSC donor, tissue source, and transfection reagent, thereby increasing the translatability of these strategies to other nonviral gene delivery systems. The work reported here demonstrates that efficient expression of two transgenes simultaneously in hMSCs can be achieved, which can greatly advance the therapeutic relevance of hMSC-based therapies for complex diseases.

## Materials and methods

### Cell culture

Cryopreserved human mesenchymal stem cells (hMSCs) from four human donors and two tissue sources were purchased at passage two from Lonza (Lonza, Walkersville, MD) and were used at passages 4, 5, and 6 (see Table S1 for donor information). Adipose-derived hMSCs (hAMSCs) were positive for CD13, CD29, CD44, CD73, CD90, CD105, CD166, and negative for CD14, CD31, CD45 cell surface markers. Bone marrow-derived hMSCs (hBMSCs) were positive for CD29, CD44, CD105, CD166 and negative for CD14, CD34, CD45 cell surface markers. hMSCs were passaged and cultured in hMSC media, consisting of Minimum Essential Medium Alpha (MEM Alpha) (Gibco, Grand Island, NY) supplemented with 10% heat-inactivated Fetal Bovine Serum (FBS) (Gibco), 6 mM L-Glutamine (Gibco), and 1% Penicillin-Streptomycin (Pen-Strep) (10,000 U/mL) (Gibco), and incubated at 37˚C with 5% CO_2_ until confluent. At confluence, hMSC media was removed and cells were washed with 1X phosphate-buffered saline (PBS) prior to the addition of 0.25% trypsin-ethylenediamine tetraacetic acid (EDTA) (Gibco) for cellular dissociation. After dissociation, an equal volume of hMSC media was added and total cellular suspension was removed for subsequent cell pelleting via centrifugation to remove trypsin-EDTA. Cells were resuspended in warm hMSC media and counted via trypan blue exclusion using a hemocytometer prior to diluting in hMSC media for seeding, as described next.

For seeding of hAMSCs, cells were dissociated and counted, as described above, and 100 µl of 4.5 × 10^4^ cells/ml cell suspension (4,500 cells/well) (Donor 1 & 2; D1 & D2) was added to each well of clear bottom, black walled, 96-well plates (Corning Life Sciences, Corning, NY). Immediately following seeding, plates were incubated at 37˚C and 5% CO_2_ and allowed to culture for 24 h prior to transfection. For seeding of hBMSCs cells were dissociated and counted as described above, and 100 µl of 6 × 10^4^ cells/ml cell suspension (6,000 cells/well) (D3 & D4) was added to each well of clear bottom, black walled, 96-well plates (Corning Life Sciences). Immediately following seeding, plates were incubated at 37˚C and 5% CO_2_ and allowed to culture for 24 h prior to transfection. The different seeding densities for hAMSCs (4.5 × 10^4^cells/ml) and hBMSCs (6 × 10^4^ cells/ml) were selected so all experimental conditions were at ~ 80% confluence before transfection, as described below.

### DNA vector production

Single transgene DNA vectors were cloned using restriction enzyme digestion and ligation. pEGFP was cloned from pEGFPLuc-CMV (Clontech, Mountain View, CA) by first linearizing with PCR (see Table S2 for primer sequences) and then restriction digesting with HindIII prior to ligation. tdTomato was cloned into pEGFP using restriction digestion with AgeI and BsrGI prior to ligation, thereby replacing EGFP with tdTomato to generate ptdTomato. Bi-cistronic DNA vectors were cloned using NEBuilder HiFi DNA assembly (New England Biolabs, Ipswich, MA). pcDNA5-MTS-TagBFP-P2AT2A-EGFP-NLS-P2AT2A-mCherry-PTS1 was a gift from Andrea Musacchio (Addgene plasmid # 87,829; http://n2t.net/addgene:87829; RRID:Addgene_87829) [22] and was used to clone the bi-cistronic pD2A (Fig. 1a). pcDNA3-TDsmURFP-IRES-eGFP was a gift from Erik Rodriguez and Roger Tsien (Addgene plasmid # 80,344; http://n2t.net/addgene:80344; RRID:Addgene_80344) [49] and was used to clone the bi-cistronic pIRES (Fig. 1). All DNA vectors were propagated in DH5α *E. coli*. (Invitrogen, Carlsbad, CA) under kanamycin selection, and isolated and purified using a Purelink High Purity Endotoxin free plasmid purification kit (Invitrogen). DNA quality and yield were measured using a Nanodrop (Thermo Fisher Scientific) and all DNA vectors were resuspended in Tris EDTA (TE) buffer at a concentration of 1 µg/µl. The bi-cistronic plasmids pEGFP_D2A_tdTomato (Addgene plasmid # 184,045) and pEGFP_IRES_tdTomato (Addgene plasmid # 184,046) cloned for this study were deposited to Addgene plasmid repository and are available for distribution at https://www.addgene.org/Angela_Pannier/.

### hMSC transfection

Twenty-four hours after seeding of hMSCs, as described above, all DNA vectors were complexed with Lipofectamine 3000 (Invitrogen) at a DNA(µg):lipid(µl) ratio of 1:2 in serum free Opti-MEM media (Invitrogen) following the manufacturer’s protocol or complexed with Turbofect (Thermo Fisher Scientific) at a DNA(µg):polymer(ul) ratio of 1:4 in serum free Opti-MEM media following manufacturer’s protocol. After complex formation, 0.07 µg of DNA vector complexed with Lipofectamine 3000 (in 6.7 µl of Opti-MEM) or 0.07 µg of DNA vector complexed with Turbofect (in 11.1 µl of Opt-MEM) was delivered to each well, and plates were briefly centrifuged to ensure mixing of complexes with the hMSC media [50]. Media was removed immediately after centrifugation and replaced with fresh, warmed, hMSC media lacking complexes. To compare DNA vectors, an equal amount of transgene (i.e., molarity of expression cassette) and DNA mass was delivered to hMSCs for each DNA vector. When directly comparing delivery of two DNA vectors to delivery of a single DNA vector or the bi-cistronic DNA vectors, one half volume of an expressionless plasmid (i.e., promoter removed from the expression cassette of pEGFP) was mixed with the single DNA vector conditions and the bi-cistronic DNA vector conditions to equalize the copy number of each transgene being delivered, as well as the total mass of DNA. Following complex delivery, hMSCs were placed into incubators at 37 °C and 5% CO_2_ and allowed to culture for 24 h prior to transfection assessment.

### Cell staining and high content imaging for transfection efficiency assessment

Twenty-four hours after delivery of complexes, cells were stained with Hoechst 33342 (Sigma-Aldrich, St. Louis, MO) to enable nuclei counts for assessment of EGFP and tdTomato transfection efficiencies. For staining, 50 µl of 3x staining solution (3 µg/ml of Hoechst in hMSC media) was added to each well and incubated for 25 min at 37˚C and 5% CO2. After incubation, staining solution was removed, and cells were rinsed with 20 µl of 1X PBS on a multi-purpose rotator for 5 min, after which the rinse was removed and 100 µl of 1X PBS was added to each well for subsequent imaging.

Images of each well were acquired with a Cytation 1 Cell Imaging System (Biotek, Winooski, VT), equipped with a laser autofocus cube and GFP (excitation 469, emission 525; EGFP transgene production), RFP (excitation 531, emission 593; tdTomato transgene production) and DAPI (excitation 377, emission 447; nuclei count via Hoechst) filter cubes paired with 465 nm, 523 nm, and 365 nm LED cubes, respectively. Two images, spaced 150 μm apart vertically, were taken of each well in each fluorescent channel, in addition to phase contrast images, using a 4x objective. Consistent fluorescence excitation LED intensity and camera exposure settings were used to allow for comparison of image intensities between wells in the same plate.

### Assessment of transfection efficiency and transgene expression levels

Gen5 software (Biotek) was used for image preprocessing (to subtract background fluorescence from captured digital images), as well as object analysis (i.e., EGFP and tdTomato positive cells, as well as cell nuclei) to calculate transfection efficiencies. Object analysis identified objects of interest in all channels by their fluorescence intensity and size. GPF, RFP, and DAPI intensity thresholds were set at 2500, 2500, and 5000 relative fluorescent units (RFU), respectively, and minimum and maximum object size set at 15 and 1000 μm (GFP and RFP) and 12 and 50 μm (DAPI).

Transfection efficiency was calculated by dividing the number of EGFP or tdTomato positive objects (cells producing transgene) by the number of DAPI objects (cell nuclei) in the same well. Co-expression was calculated by adding a secondary filter to the EGFP positive objects to identify EGFP positive objects that were also tdTomato positive with an RFU greater than 2500. This number of co-expressing cells was then divided by DAPI objects in the same well to calculate the transfection efficiency of co-expressing cells, or divided by the number of EGFP positive cells to calculate the percentage of transfected cells that were expressing both transgenes. See Figure S1 for representative images of transfected cells used in the analysis.

### Data analysis and statistics

All data are reported as the mean of triplicate values for each condition on duplicate days (*n* = 6), except where noted. Transfection efficiencies were analyzed using a 2-way ANOVA with Tukey’s post hoc test or using a two-tailed T-test. Adjusted *p*-values were calculated as multiplicity adjusted *p*-values [51]. Statistical significance was accepted for *p*-values less than 0.05. Statistics were evaluated using Prism GraphPad software (GraphPad Software, Inc, La Jolla, CA).

### Supplementary Information

**Additional file 1: Table S1.** Information on hMSC Donors Used in Transfection Studies. Word table with hMSC donor ID, tissue source, age, sex, and ethnicity/race for all hMSC donors used in this study.

**Additional file 2: Table S2.** Primers used for Plasmid Cloning. Word table with primer ID, sequence, and the plasmid names the primers were used for to clone.

**Additional file 3: Figure S1.** Expression of Two Transgenes in hMSCs. Representative images of all conditions tested to express two transgenes in D1 hAMSCs.  a) Overlaid fluorescent and brightfield images of EGFP (green cells), tdTomato (red cells), co-expressing (yellow cells), and untransfected cells (light grey cells) for delivery of two DNA vectors delivered in separate complexes using Lipofectamine 3000 as the cationic carrier,  b) for delivery of two DNA vectors delivered in the same complex using Lipofectamine 3000 as the cationic carrier,  c) for delivery of a bi-cistronic IRES DNA vector using Lipofectamine 3000 as the cationic carrier,  d) for delivery of a bi-cistronic D2A DNA vector using Lipofectamine 3000 as the cationic carrier, e) for delivery of two DNA vectors delivered in separate complexes using Turbofect as the cationic carrier,  f) for delivery of two DNA vectors delivered in the same complex using Turbofect as the cationic carrier,  g) for delivery of a bi-cistronic IRES DNA vector using Turbofect as the cationic carrier, and  h) for delivery of a bi-cistronic D2A DNA vector using Turbofect as the cationic carrier.  Scale bar is 1000 µm.

## Data Availability

The data sets used and/or analyzed during the current study are available from the corresponding author on reasonable request.
