# Supplementary material for: Systematic comparison of nonviral gene delivery strategies for efficient co-expression of two transgenes in human mesenchymal stem cells
Source: J Biol Eng. 2023 Dec 7;17:76. doi: 10.1186/s13036-023-00394-0 (PMC10704746; doi:10.1186/s13036-023-00394-0)
Supplement: Supplementary file 1 — Additional file 1: Table S1. Information on hMSC Donors Used in Transfection Studies. Word table with hMSC donor ID, tissue source, age, sex, and ethnicity/race for all hMSC donors used in this study. [file 13036_2023_394_MOESM1_ESM.docx]

Table S1: Information on hMSC Donors Used in Transfection Studies

| Doner ID | Tissue Source | Age | Sex | Ethnicity/Race |
| --- | --- | --- | --- | --- |
| D1 | Adipose | 30 | F | Hispanic |
| D2 | Adipose | 23 | F | Black |
| D3 | Bone Marrow | 22 | M | Not Provided |
| D4 | Bone Marrow | 26 | M | Black |
