# Supplementary material for: Systematic comparison of nonviral gene delivery strategies for efficient co-expression of two transgenes in human mesenchymal stem cells
Source: J Biol Eng. 2023 Dec 7;17:76. doi: 10.1186/s13036-023-00394-0 (PMC10704746; doi:10.1186/s13036-023-00394-0)
Supplement: Supplementary file 2 — Additional file 2: Table S2. Primers used for Plasmid Cloning. Word table with primer ID, sequence, and the plasmid names the primers were used for to clone. [file 13036_2023_394_MOESM2_ESM.docx]

| Primer ID | Primer Sequence | Plasmid |
| --- | --- | --- |
| ME1_HindIII | CCGCTGAAGCTTTACTTGTACAGCTCGTCCATGC | pEGFP |
| ME2Full_HindIII | CCGGGCAAGCTTAATTCTCGAAGATCCACCGGATCTA |  |
| TDA_Vector.FOR | CTGTACAAGTAGTAAAGCTTAATTCTCGAAGATCCACCGG | pD2A |
| TDA_Vector.REV | TGCCTCCGGACTTGTACAGCTCGTCCATGC |  |
| TDA_Frag_1.FOR | GCTGTACAAGTCCGGAGGCAGAAAGCTTGGTTCC |  |
| TDA_Frag_1.REV | TGCTCACCATGGTAGATCCGAG |  |
| TDA_Frag_2.FOR | CGGATCTACCATGGTGAGCAAGGGCGA |  |
| TDA_Frag_2.REV | GAATTAAGCTTTACTACTTGTACAGCTCGTCCATGC |  |
| TI_Vector.FOR | TGTACAAGTAGAGCTTAATTCTCGAAGATCCACCG | pIRES |
| TI_Vector.REV | GAGTACTCGAGTTACTTGTACAGCTCGTCCATGCCGAG |  |
| TI_Frag_1.FOR | GTACAAGTAACTCGAGTACTCCGGTATTGCGG |  |
| TI_Frag_1.REV | CCATGGTGGCCGTACGGATCCTATCCAATTCGC |  |
| TI_Frag_2.FOR | GATCCGTACGGCCACCATGGTGAGCAAGG |  |
| TI_Frag_2.REV | GAGAATTAAGCTCTACTTGTACAGCTCGTCCATGC |  |

Table S2: Primers used for Plasmid Cloning
