# Supplementary figures and images for: Systematic comparison of nonviral gene delivery strategies for efficient co-expression of two transgenes in human mesenchymal stem cells
Source: J Biol Eng. 2023 Dec 7;17:76. doi: 10.1186/s13036-023-00394-0 (PMC10704746; doi:10.1186/s13036-023-00394-0)

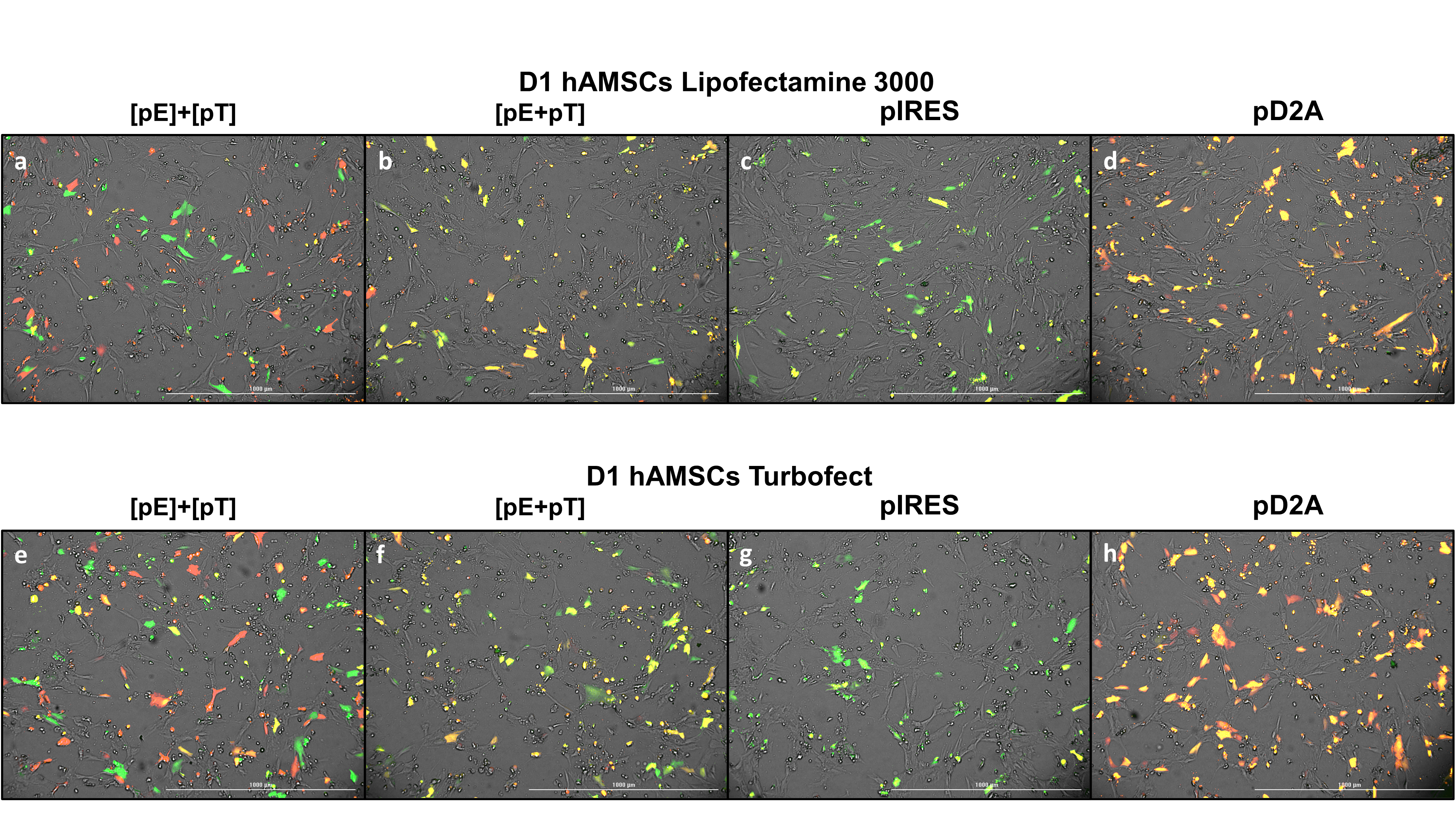

Supplement: Supplementary file 3 — Additional file 3: Figure S1. Expression of Two Transgenes in hMSCs. Representative images of all conditions tested to express two transgenes in D1 hAMSCs. a) Overlaid fluorescent and brightfield images of EGFP (green cells), tdTomato (red cells), co-expressing (yellow cells), and untransfected cells (light grey cells) for delivery of two DNA vectors delivered in separate complexes using Lipofectamine 3000 as the cationic carrier, b) for delivery of two DNA vectors delivered in the same complex using Lipofectamine 3000 as the cationic carrier, c) for delivery of a bi-cistronic IRES DNA vector using Lipofectamine 3000 as the cationic carrier, d) for delivery of a bi-cistronic D2A DNA vector using Lipofectamine 3000 as the cationic carrier, e) for delivery of two DNA vectors delivered in separate complexes using Turbofect as the cationic carrier, f) for delivery of two DNA vectors delivered in the same complex using Turbofect as the cationic carrier, g) for delivery of a bi-cistronic IRES DNA vector using Turbofect as the cationic carrier, and h) for delivery of a bi-cistronic D2A DNA vector using Turbofect as the cationic carrier. Scale bar is 1000 µm. [file 13036_2023_394_MOESM3_ESM.png]
